# Draft genomes of 53 *Legionella pneumophila* isolates from groundwater wells in Southern Nevada

**DOI:** 10.1128/mra.01349-25

**Published:** 2026-06-11

**Authors:** Phillip Wang, Christina M. Morrison, Daniel Gerrity

**Affiliations:** 1 Southern Nevada Water Authority 235127, Las Vegas, Nevada, USA; 2 Department of Civil and Environmental Engineering and Construction, University of Nevada Las Vegas, Las Vegas, Nevada, USA; University of Manitoba 8664, Winnipeg, Manitoba, Canada

**Keywords:** *Legionella* pneumophila, waterborne pathogens, groundwater

## Abstract

We sequenced 53 *Legionella pneumophila* isolates from Southern Nevada groundwater, an understudied source despite its use for drinking water. Genomes averaged 3.48 Mbp, and 25 carried plasmids. These data provide a resource for identifying genomic features distinguishing environmental strains from those associated with disease outbreak.

## ANNOUNCEMENT

*Legionella pneumophila*, first recognized after a 1976 outbreak in Philadelphia (1), is an opportunistic bacterial pathogen that can cause atypical pneumonia. It is ubiquitously found in water environments, and although susceptible to disinfection (2, 3), it is detected in premise plumbing systems where it replicates within biofilm-associated protozoan hosts (4, 5). *L. pneumophila* is commonly detected in groundwater (6), which is a major drinking water source for many public water systems and private wells.

Fifty-three *L. pneumophila* isolates were obtained through groundwater monitoring in Las Vegas, Nevada, USA. Groundwater samples (100 mL) were analyzed using Legiolert (IDEXX), and liquid aliquots from positive Legiolert trays (post 7-day incubation at 37°C) were streaked onto buffered charcoal yeast extract agar (Remel) with 0.2 g/L L-cysteine monohydrochloride (Millipore) and incubated for 2–5 days at 37°C. Single colonies were confirmed as *L. pneumophila* using the MALDI Biotyper Sirius (Bruker Scientific) (7, 8) using the direct smear method and analyzing mass spectra against the Bruker BDAL Library. Serogroups were broadly determined (1 vs 2–14) using latex agglutination (Oxoid). Confirmed single *L. pneumophila* colonies were enriched in buffered yeast extract broth (10 g yeast extract [Fisher Scientific], 10 g ACES buffer [Fisher Scientific], 1 g α-ketoglutaric acid [Thermo Scientific], 0.25 g ferric pyrophosphate [Spectrum], 0.4 g L-cysteine monohydrochloride [Millipore]) for 24 h at 37°C. DNA was extracted from enriched broth using the Monarch Genomic DNA Purification Kit (New England Biolabs), per manufacturer’s instructions. DNA concentrations were determined using the Qubit HS DNA assay kit (Thermo Fisher Scientific).

Sequencing libraries were prepared with the Rapid Barcoding Kit 24 v14 (SQK-RBK114.24) (Oxford Nanopore Technologies [ONT]) and sequenced using the MinION MK1b platform (ONT) with a FLO-MIN114 R10.4.1 flow cell (ONT) for 72 h, per manufacturer’s instructions. A total of 4.23 million reads were generated across all samples (average read length of 7.5 kb). Base calling was performed with Dorado v1.1.1 (9) in super-accurate mode. Adapters were removed, and read quality was filtered with a phred score of 9 using NanoFilt v2.8 (10). Draft genomes were *de novo* assembled using Flye v2.9.5 (11) specified for high quality ONT reads (<5% error rate). Assembled genomes were polished with Medaka v1.12.1 (ONT, https://github.com/nanoporetech/medaka) using default settings. Assembly metrics were evaluated using QUAST v5.3 (12) with standard settings. The majority of isolates resulted in a single chromosomal contig, and a subset of these were identified as circular by Flye v2.9.5. Genome rotation to a standardized origin dnaA was performed. Plasmid-associated contigs were identified using the mob_recon module of MOB-suite v3.1.9 (13), which reconstructs and types plasmids from draft assemblies based on known replicon and mobilization (MOB) genes.

Mean GC content was 38.35% ± 0.05% for chromosomes and 37.95% ± 0.93% for plasmids. None of the 53 isolates had the *lag-1* gene, aligning with prior studies showing that *lag-1* is present in 70%–90% of clinical or outbreak-associated strains but in only a small minority of environmental isolates (14, 15). An overview of the 53 *L. pneumophila* groundwater isolates is provided in Table 1.

**TABLE 1:** Overview of genome assemblies from 53 *L. pneumophila* isolates

NCBI accession number (draft genome)	NCBI SRA number	Isolate name	Serogroup	Mean genome coverage	Chromosome scaffolds	Chromosome scaffold sizes (bp)	GC%	Plasmid number	Plasmid size (bp)	GC%	Relaxase type
JBTJEK000000000	SRX32614527	Env01	2–14	249	1	3,453,717	38.35	0			
JBTJEJ000000000	SRX32614528	Env03	1	153	1	3,519,436	38.36	1	99,671	40.14	MOBP
JBTJEI000000000	SRX32614539	Env04	2–14	469	1	3,411,208	38.35	0			
JBTJEH000000000	SRX32614550	Env07	1	41	1	3,512,589	38.37	0			
JBTJEG000000000	SRX32614561	Env13	2–14	63	1	3,632,815	38.36	0			
JBTJEF000000000	SRX32614572	Env14	2–14	60	1	3,621,368	38.31	0			
JBTJEE000000000	SRX32614576	Env16	2–14	92	1	3,448,787	38.29	0			
JBTJED000000000	SRX32614577	Env17	1	75	1	3,495,839	38.35	1	150,922	37.64	MOBF
JBTJEC000000000	SRX32614578	Env18	2–14	243	1	3,427,839	38.34	0			
JBTJEB000000000	SRX32614579	Env19	2–14	106	2	3,685,559, 174,601	38.35, 39.75	0			
JBTJEA000000000	SRX32614529	Env21	2–14	219	1	3,435,846	38.30	0			
JBTJDZ000000000	SRX32614530	Env22	1	156	1	3,495,876	38.32	1	129,879	37.45	MOBF
JBTJDY000000000	SRX32614531	Env24	2–14	153	1	3,550,733	38.33	1	62,455	37.00	MOBF
JBTJDX000000000	SRX32614532	Env33	2–14	197	1	3,427,849	38.35	0			
JBTJDW000000000	SRX32614533	Env34	2–14	149	2	3,685,559, 174,594	38.35, 39.17	0			
JBTJDV000000000	SRX32614534	Env35	1	232	1	3,488,019	38.35	1	129,882	37.45	MOBF
JBTJDU000000000	SRX32614535	Env36	2–14	221	1	3,523,366	38.39	0			
JBTJDT000000000	SRX32614536	Env37	1	135	1	3,489,676	38.36	1	129,872	37.45	MOBF
JBTJDS000000000	SRX32614537	Env38	1	81	1	3,495,895	38.35	1	129,883	37.45	MOBF
JBTJDR000000000	SRX32614538	Env39	2–14	199	1	3,455,011	38.30	0			
JBTJDQ000000000	SRX32614540	Env42	1	135	1	3,488,012	38.35	1	129,880	37.46	MOBF
JBTJDP000000000	SRX32614541	Env43	2–14	113	1	3,402,582	38.36	0			
JBTJDO000000000	SRX32614542	Env46	2–14	231	1	3,427,831	38.33	0			
JBTJDN000000000	SRX32614543	Env47	1	351	1	3,574,283	38.38	0			
JBTJDM000000000	SRX32614544	Env48	1/2–14^*b*^	57	1	3,411,217	38.34	0			
JBTJDL000000000	SRX32614545	Env50	2–14	281	1	3,418,391	38.38	0			
JBTJDK000000000	SRX32614546	Env51	2–14	324	1	3,435,832	38.30	0			
JBTJDJ000000000	SRX32614547	Env52	1	304	1	3,489,114	38.35	1	129,873	37.45	MOBF
JBTJDI000000000	SRX32614548	Env53	1	328	1	3,581,876	38.37	1	129,871	37.45	MOBF
JBTJDH000000000	SRX32614549	Env54	1	185	1	3,493,638	38.35	1	129,875	37.45	MOBF
JBTJDG000000000	SRX32614551	Env55	No RXN^*a*^	83	1	3,471,973	38.35	1	61,986	39.32	MOBF
JBTJDF000000000	SRX32614552	Env56	1	319	1	3,519,440	38.65	1	99,666	40.14	MOBP
JBTJDE000000000	SRX32614553	Env57	1	130	1	3,489,580	38.35	1	129,865	37.45	MOBF
JBTJDD000000000	SRX32614554	Env58	1	210	1	3,495,895	38.35	1	129,869	37.45	MOBF
JBTJDC000000000	SRX32614555	Env59	2–14	194	1	3,435,818	38.29	0			
JBTJDB000000000	SRX32614556	Env60	1	213	1	3,495,883	38.35	1	129,874	37.45	MOBF
JBTJDA000000000	SRX32614557	Env61	1	134	1	3,494,293	38.35	1	129,874	37.45	MOBF
JBTJCZ000000000	SRX32614558	Env62	2–14	20	2	3,447,794 6124	38.28, 36.98	0			
JBTJCY000000000	SRX32614559	Env63	2–14	118	1	3,428,694	38.29	1	86,832	37.62	MOBF_MOBQ
JBTJCX000000000	SRX32614560	Env64	1/2–14^*b*^	139	1	3,471,975	38.35	1	79,289	39.49	MOBF
JBTJCW000000000	SRX32614562	Env65	2–14	151	2	3,685,587, 174,598	38.35, 39.17				
JBTJCV000000000	SRX32614563	Env66	2–14	35	1	3,427,826	38.33	0			
JBTJCU000000000	SRX32614564	Env67	1	131	1	3,495,003	38.41	0			
JBTJCT000000000	SRX32614565	Env68	1	32	1	3,484,722	38.34	1	129,874	37.45	MOBF
JBTJCS000000000	SRX32614566	Env69	2–14	147	1	3,416,673	38.37	0			
JBTJCR000000000	SRX32614567	Env70	2–14	221	1	3,498,314	38.40	0			
JBTJCQ000000000	SRX32614568	Env71	2–14	134	1	3,370,598	38.27	1	59,462	38.25	MOBF
JBTJCP000000000	SRX32614569	Env72	2–14	156	1	3,523,390	38.39	0			
JBTJCO000000000	SRX32614570	Env73	1	169	1	3,488,012	38.35	1	129,870	37.45	MOBF
JBTJCN000000000	SRX32614571	Env74	1	191	1	3,472,071	38.33	0			
JBTJCM000000000	SRX32614573	Env101	1	625	1	3,489,659	38.35	1	129,878	37.45	MOBF
JBTJCL000000000	SRX32614574	En102	1	145	1	3,493,492	38.35	1	129,869	37.45	MOBF
JBTJCK000000000	SRX32614575	Env103	1	35	1	3,489,653	38.35	1	129,869	37.45	MOBF

^*a*^ No reaction with latex agglutination kit but confirmed *L. pneumophila* via MALDI-MS.

^*b*^ Sample reacted with SG1 latex agglutination test but was determined to likely be SG2-14 based on genomic characterization.

## Data Availability

Raw reads and genome assemblies are deposited in NCBI BioProject PRJNA1357352; accession/SRA numbers are provided in Table 1.
